# Supplementary material for: Content-rich biological network constructed by mining PubMed abstracts
Source: BMC Bioinformatics. 2004 Oct 8;5:147. doi: 10.1186/1471-2105-5-147 (PMC528731; doi:10.1186/1471-2105-5-147)
Supplement: Additional File 2 — The original results of the above study (non-essential files are deleted to keep the file size under the limit set by BMC bioinformatics). [file 1471-2105-5-147-S2.bz2 › chilibotAdditionalFile2/dip20/4ID9194565E6/html/P300_TP53.html]

 


 **P300** and **TP53** 
  
Found 17 abstracts in PubMed, retrieved 17.  
 

 What does Google say? 
 PDF only 
| .edu only 

---

**Interactive relationship** (e.g. stimulation, inhibition, etc)

**Inhibitory relationship**- The tumor suppressor p53  [ **TP53** ]  recruits the cellular coactivator CBP  **p300**  to mediate the transcriptional activation of target genes.  Ref: 11782467 J Biol Chem, 2002
- **p300**  acetylates and activates the tumor suppressor p53  [ **TP53** ]  after DNA damage.  Ref: 11070080 Proc Natl Acad Sci U S A, 2000
**Neutral relationship**

**Non-interactive relationship** (e.g. studied together, co-existance, homology, etc.)

- Known targets of the 708 amino acid large T antigen include the three members of the retinoblastoma protein family pRb, p107, and p130, members of the CBP family of transcriptional adapter proteins cap binding protein CBP,  **p300** , and p400, and the tumor suppressor p53  [ **TP53** ] .  Ref: 9234732 Mol Cell Biol, 1997
- Our previous study shows that MDM2, a negative feedback regulator of the tumor suppressor p53  [ **TP53** ] , inhibits  **p300**  mediated p53 acetylation.  Ref: 12068014 J Biol Chem, 2002
